# Changes in Metabotropic Glutamate Receptor Gene Expression in Rat Brain in a Lithium–Pilocarpine Model of Temporal Lobe Epilepsy

**DOI:** 10.3390/ijms23052752

**Published:** 2022-03-02

**Authors:** Anna A. Kovalenko, Maria V. Zakharova, Alexander P. Schwarz, Alexandra V. Dyomina, Olga E. Zubareva, Aleksey V. Zaitsev

**Affiliations:** Laboratory of Molecular Mechanisms of Neural Interactions, Sechenov Institute of Evolutionary Physiology and Biochemistry of RAS, 194223 Saint Petersburg, Russia; kovalenko_0911@mail.ru (A.A.K.); zaharova-masha@yandex.ru (M.V.Z.); aleksandr.pavlovich.schwarz@gmail.com (A.P.S.); adyomina513@gmail.com (A.V.D.); zubarevaoe@mail.ru (O.E.Z.)

**Keywords:** temporal lobe epilepsy, metabotropic glutamate receptor, RT-qPCR, lithium–pilocarpine model, hippocampus, temporal cortex

## Abstract

Preventing epileptogenesis in people at risk is an unmet medical need. Metabotropic glutamate receptors (mGluRs) are promising targets for such therapy. However, drugs acting on mGluRs are not used in the clinic due to limited knowledge of the involvement of mGluRs in epileptogenesis. This study aimed to analyze the changes in gene expression of mGluR subtypes (1–5, 7, 8) in various rat brain regions in the latent and chronic phases of a lithium–pilocarpine model of epilepsy. For this study, multiplex test systems were selected and optimized to analyze mGluR gene expression using RT-qPCR. Region- and phase-specific changes in expression were revealed. During the latent phase, mGluR5 mRNA levels were increased in the dorsal and ventral hippocampus, and expression of group III genes was decreased in the hippocampus and temporal cortex, which could contribute to epileptogenesis. Most of the changes in expression detected in the latent stage were absent in the chronic stage, but mGluR8 mRNA production remained reduced in the hippocampus. Moreover, we found that gene expression of group II mGluRs was altered only in the chronic phase. The study deepened our understanding of the mechanisms of epileptogenesis and suggested that agonists of group III mGluRs are the most promising targets for preventing epilepsy.

## 1. Introduction

Epilepsy is a chronic neurological disorder that affects millions of people [1]. Standard antiseizure medications fail to control seizures in about a fifth to a third of patients [2]. Since up to 40% of cases are an acquired form of epilepsy, prevention of epilepsy in patients at risk is a primary unmet medical need [3]. The difficulties in preventing epilepsy are due to the lack of knowledge of the pathogenetic mechanisms of its development. An imbalance between excitatory and inhibitory neurotransmission is one of the critical mechanisms of epileptogenesis [4,5]. Metabotropic glutamate receptors (mGluRs) modulate excitatory and inhibitory synaptic transmission and plasticity [6,7]. Thus, mGluRs are essential in maintaining the excitation/inhibition balance [8]; moreover, they have been shown to be directly involved in epileptogenesis [9].

Different types of mGluRs are present on the presynaptic and postsynaptic membrane of neurons and glial cells, providing subtle regulation of signal transmission [10]. There are three groups of mGluRs, which include eight subtypes based on sequence homology, G-protein binding, and ligand selectivity [10].

Group I includes mGluR1 and mGluR5 coupled to G_q_/G_11_ proteins. They are expressed mainly postsynaptically, and their activation is associated with the stimulation of the phospholipase C pathway. Initiation of this pathway leads to hydrolysis of phosphatidylinositol-4,5-bisphosphate to form inositol-1,4,5-trisphosphate and diacylglycerol, which increase intracellular Ca^2+^ release and activate protein kinase C, respectively [6]. Activation of receptors in this group may contribute to the activation of glutamate N-methyl-D-aspartate (NMDA) receptors. 

Group II includes mGluR2 and mGluR3 coupled to G_i_/G_o_ proteins; mGluR2 has a predominantly presynaptic localization, whereas mGluR3 can be located postsynaptically and on the presynapse, where it interacts with mGluR5 [10,11]. The effects of their activation are to inhibit adenylate cyclase and Ca^2+^ channels and activate K^+^ channels [10]. The primary function of the presynaptic group II receptors is to inhibit neurotransmitter release [8]. In addition, mGluR3 is expressed in glial cells.

Group III includes mGluR4, mGluR6, mGluR7, and mGluR8, which are also linked to G_i_/G_o_ proteins and inhibit neurotransmitter release [6]—mGluR4, mGluR7, and mGluR8 are localized presynaptically at the active zone of neurotransmitter release [8], while mGluR6 is expressed in retinal cells; therefore, we did not consider this subtype in the paper.

The effectiveness of mGluR ligands in the treatment of neurological diseases such as Alzheimer’s disease, Parkinson’s disease, anxiety, depression, schizophrenia, and epilepsy was supported by recent studies [6,10]. However, it is unclear whether mGluR ligands can be used in the clinic to prevent epilepsy development, due to the limited data on mGluR gene expression at different stages of epileptogenesis [9].

It is known that prolonged disruption of mGluR gene expression can lead to impaired synaptic plasticity, increased glutamate concentrations, and overexcitation in epilepsy [6]. Group I mGluR production was enhanced in the hippocampus both in patients with epilepsy and in rats after seizures induced by electrical stimulation [12,13,14]. A study by Aronica et al. also found upregulation of mGluR3 expression in astrocytes of rats in a model of temporal lobe epilepsy (TLE) [12]. Another study revealed a significant decrease in mGluR2 and mGluR3 expression and function of both subtypes in the dentate gyrus of the hippocampus and cortex in a pilocarpine model of chronic epilepsy 24 h after induction of seizures [15]. However, there is a lack of data on group III mGluR expression in rat brains after seizures. 

Overall, changes in mGluR gene expression and the role of these changes in epileptogenesis still remain poorly understood. Therefore, this study aimed to analyze changes in gene expression of all groups of mGluRs in the dorsal and ventral regions of rat hippocampus and temporal cortex during epileptogenesis and the chronic phase of a lithium–pilocarpine epilepsy model.

## 2. Results

The lithium–pilocarpine model used in our study is the most relevant model of TLE [16,17]. It is characterized by the essential features of TLE, such as: (i) seizure foci in the hippocampus, entorhinal cortex, or amygdala [18]; (ii) an “initial precipitating injury” that often precedes the onset of TLE [19]; and (iii) a latent period without seizures [20]. Only rats with severe prolonged seizures (grade 4 on the Racine scale [21]) were included in the study. After 75 min, seizures were stopped by diazepam (5 mg/kg). The control and experimental groups included at least nine animals. 

We analyzed changes in the gene expression of groups I (*Grm1*, *Grm5*), II (*Grm2*, *Grm3*), and III (*Grm4*, *Grm7*, *Grm8*) mGluRs in the dorsal and ventral hippocampus, and temporal cortex in the latent (3 and 7 days after pilocarpine-induced seizures) and chronic (60 days) phases of the model using real-time RT-qPCR (TaqMan technology). 

**Figure A1:**
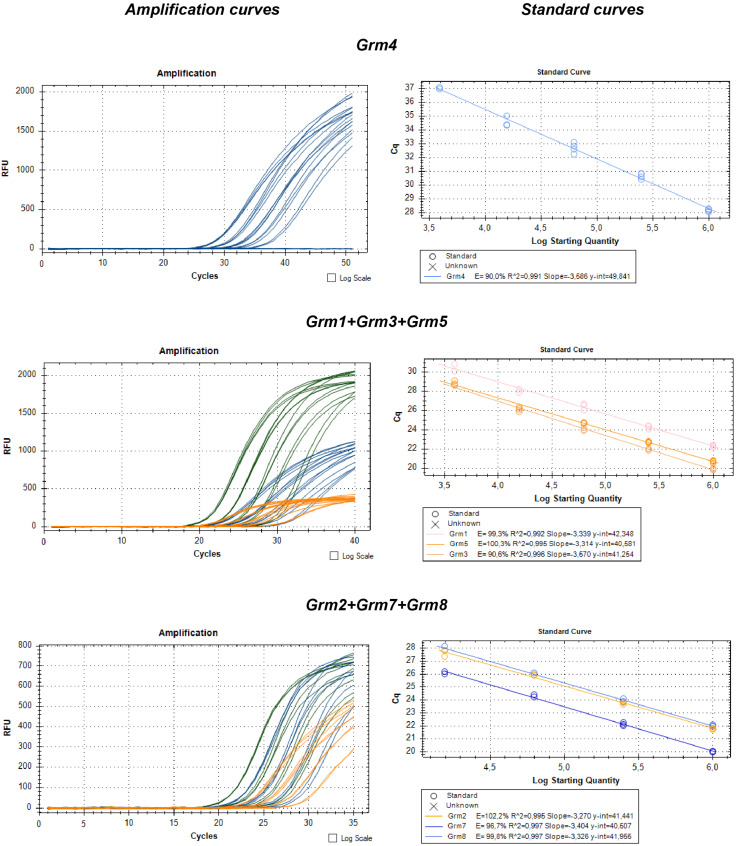
A representative experiment to test the efficiencies of PCR systems selected in this work by the serial dilution method. (4-fold dilution series). The most concentrated sample corresponded to 10-fold-diluted cDNA synthesized from 1 μg of rat temporal cortex total RNA. PCR reactions were triplicated and carried out in a C1000 Touch thermal cycler combined with a CFX384 Touch Real-Time PCR Detection System (BioRad, Hercules, CA, USA) simultaneously with no template control samples. The colors of the amplification curves indicate the detection channel (blue for FAM (*Grm4*, *Grm2*, *Grm1*), green for HEX (*Grm5*, *Grm7*), orange for ROX (*Grm3*, *Grm8*)).

Multiplex systems were selected and optimized during this study to detect different types of mGluR genes simultaneously (Figure A1). These systems allowed us to assess the relative expression of mGluR genes more quickly and accurately. We revealed the phase- and region-specific changes in the expression for most studied genes.

### 2.1. Changes in the Gene Expression of mGluRs in the Latent Phase of the Model

**Figure 1:**
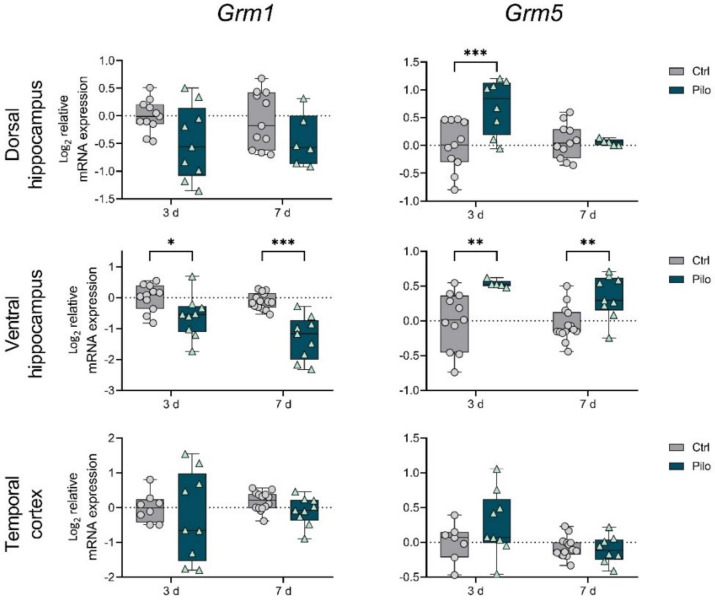
The relative mRNA expression of group I metabotropic glutamate receptors (mGluRs) *Grm1* and *Grm5* in the temporal cortex (TC), dorsal (DH), and ventral (VH) hippocampus in a lithium–pilocarpine model of temporal lobe epilepsy. The studies were performed at 3 (3 d) and 7 (7 d) days after the seizure induction (latent phase of the model). Ctrl—control group; Pilo—experimental group. After two-way ANOVA, only statistically significant effects (*p* < 0.05) are reported here: *Grm1:* VH—F_1,39_ (seizure) = 31.6, *p* < 0.001; *Grm5:* DH—F_1,31_ (seizure × day) = 6.8, *p* = 0.01, F_1,31_ (day) = 4.3, *p* = 0.04, F_1,31_ (seizure) = 6.2, *p* = 0.02; VH—F_1,35_ (seizure) = 19.7, *p* < 0.001. Asterisks indicate significant differences between groups according to Sidak’s post hoc tests: * *p* < 0.05, ** *p* < 0.01, *** *p* < 0.001. Data are represented as individual values (circles and triangles) with the minimum and maximum (error bars), the sample median (horizontal line), and the first and third quartiles (boxes).

**Figure 2:**
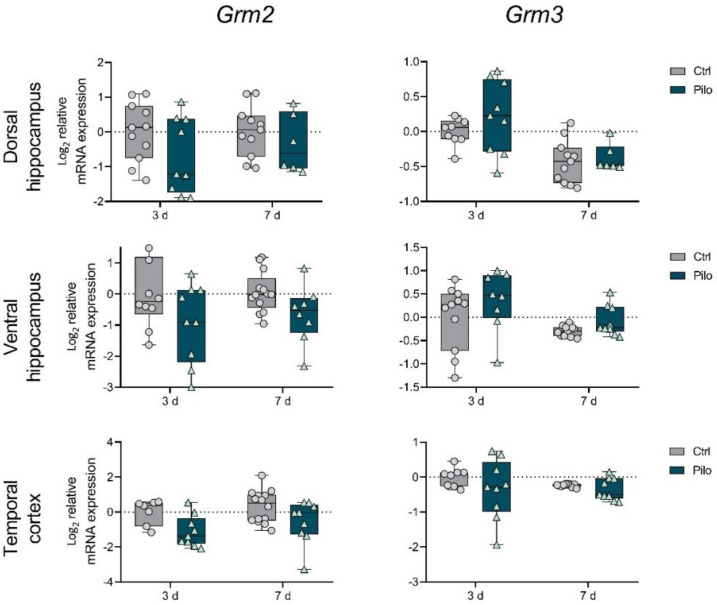
The relative mRNA expression of group II metabotropic glutamate receptors *Grm2* and *Grm3* in the temporal cortex (TC), dorsal (DH), and ventral (VH) hippocampus in a lithium–pilocarpine model of temporal lobe epilepsy. The studies were performed at 3 (3 d) and 7 (7 d) days after the seizure induction (latent phase of the model). Ctrl—control group; Pilo—experimental group. After two-way ANOVA, only statistically significant effects (*p* < 0.05) are reported: *Grm2:* VH—F_1,36_ (seizure) = 6.8, *p* = 0.01; *Grm3:* DH—F_1,31_ (day) = 18.8, *p* < 0.001; VH—F_1,35_ (day) = 5.1, *p* = 0.03; TC—F_1,35_ (seizure) = 9.9, *p* = 0.003. Data are represented as individual values (circles and triangles) with the minimum and maximum (error bars), the sample median (horizontal line), and the first and third quartiles (boxes).

**Figure 3:**
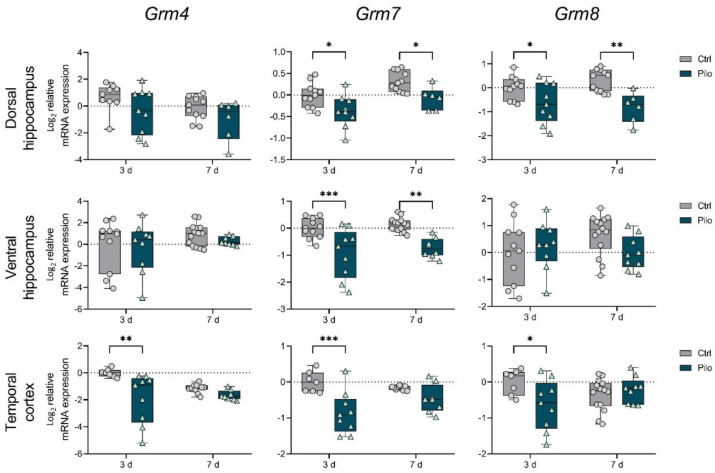
The relative mRNA expression of group III metabotropic glutamate receptors *Grm4, Grm7,* and *Grm8* in the temporal cortex (TC), dorsal (DH), and ventral (VH) hippocampus in a lithium–pilocarpine model of temporal lobe epilepsy. The studies were performed at 3 (3 d) and 7 (7 d) days after the seizure induction (latent phase of the model). Ctrl—control group; Pilo—experimental group. After two-way ANOVA, only statistically significant effects (*p* < 0.05) are reported: *Grm4:* DH—F_1,31_ (seizure) = 4.8, *p* = 0.04; TC—F_1,31_ (seizure × day) = 4.4, *p* = 0.04, F_1,31_ (seizure) = 12.7, *p* = 0.001; *Grm7:* DH—F_1,33_ (day) = 9.5, *p* = 0.004, F_1,33_ (seizure) = 14.6, *p* < 0.001; VH—F_1,38_ (seizure) = 30.8, *p* < 0.001; TC—F_1,29_ (seizure) = 18.3, *p* < 0.001; *Grm8:* DH—F_1,33_ (seizure) = 18.6, *p* < 0.001; TC—F_1,35_ (seizure × day) = 5.8, *p* = 0.02. Asterisks indicate significant differences between groups according to Sidak’s post hoc tests: * *p* < 0.05, ** *p* < 0.01, *** *p* < 0.001. Data are represented as individual values (circles and triangles) with the minimum and maximum (error bars), the sample median (horizontal line), and the first and third quartiles (boxes).

First, we detected that in the latent phase of the model, the changes in gene expression of mGluRs in groups I and III were more profound than changes in expression of group II mGluRs (Figure 1, Figure 2 and Figure 3).

In particular, a decrease in the production of *Grm1* in the ventral area of the hippocampus was observed on days 3 and 7 after seizures (Figure 1, two-way ANOVA). The mRNA level of the *Grm5* gene was upregulated in both hippocampus areas on day 3 and in only the ventral hippocampus on day 7 after seizure induction. 

Pilocarpine-induced seizures affected the *Grm2* and *Grm3* gene expression in the ventral hippocampus and temporal cortex; however, no significant intergroup differences were revealed by post hoc tests (Figure 2).

In group III, a decrease in the expression of *Grm4*, *Grm7*, and *Grm8* genes was revealed in the temporal cortex, but only on day 3 (Figure 3). A downregulation of *Grm7* and *Grm8* mRNA levels in the dorsal hippocampus was detected at both time points, while in the ventral hippocampus, only a decrease in *Grm7* expression was observed.

The detected increase in *Grm5* mRNA production and a decrease in group III members’ expression were likely to contribute to the development of epilepsy in the latent phase of the model. Under physiological conditions, *Grm5* promotes NMDA receptor activation, and its overexpression may contribute to excitotoxicity [6]. The mGluRs in group III inhibited neurotransmitter release [6], and a significant decrease in their expression during the latent phase may also contribute to hyperexcitation. At the same time, a decrease in *Grm1* expression can be considered as a compensatory mechanism.

### 2.2. Changes in the Gene Expression of mGluRs in the Chronic Phase of the Model

Next, we analyzed mGluR gene expression two months after pilocarpine administration in the chronic phase of the model. Only epileptic rats with registered spontaneous recurrent seizures were included in the experimental group (*n* = 10). 

We found that the expression of some mGluR genes was altered in epileptic animals. Changes were observed in both parts of the hippocampus and the temporal cortex. However, alterations in the expression of the genes studied were less pronounced in the chronic phase of the model than in the latent phase. Most of the changes in expression detected in the latent stage were absent in the chronic stage, except for mGluR1 and mGluR8. Moreover, we found that gene expression of group II mGluRs was altered only in the chronic phase. 

**Figure 4:**
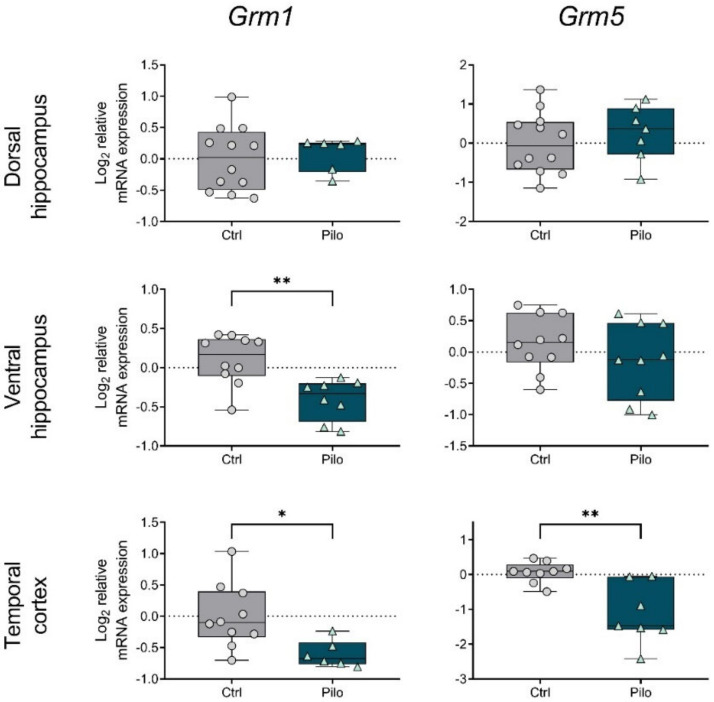
The relative mRNA expression of group I metabotropic glutamate receptors *Grm1* and *Grm5* in the temporal cortex (TC), dorsal (DH), and ventral (VH) hippocampus in a lithium–pilocarpine model of temporal lobe epilepsy. The studies were performed two months after the seizure induction (chronic phase of the model). Ctrl—control group; Pilo—epileptic animals. Data are represented as individual values (circles and triangles) with the minimum and maximum (error bars), the sample median (horizontal line), and the first and third quartiles (boxes). Unpaired *t*-test: * *p* < 0.05, ** *p* < 0.01.

In particular, a decrease in *Grm1* expression was noted in the ventral hippocampus and temporal cortex (Figure 4). In addition, a decrease in mRNA production of the *Grm5* gene was detected in the temporal cortex. The observed decrease in the expression of mGluRs from group I could be a compensatory mechanism to reduce hyperexcitation.

**Figure 5:**
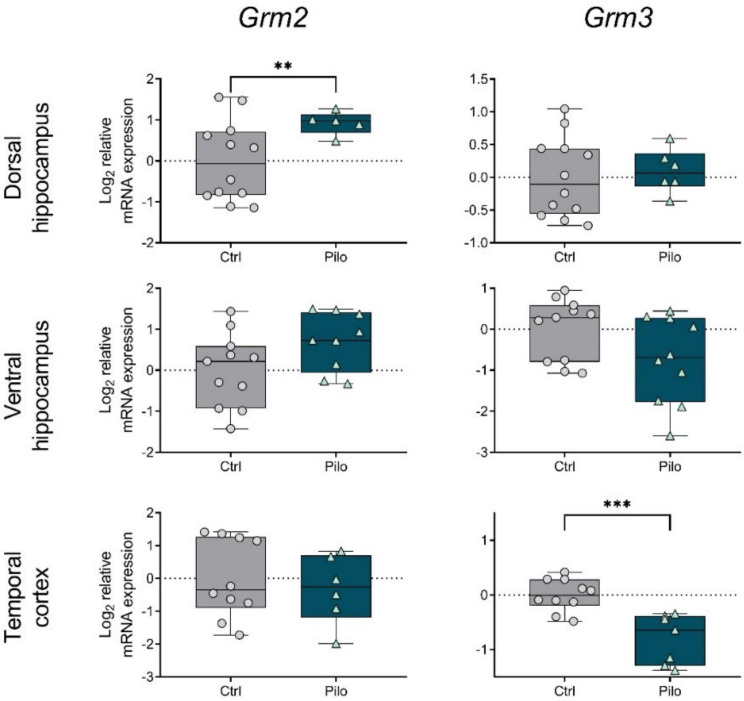
The relative mRNA expression of group II metabotropic glutamate receptors *Grm2* and *Grm3* in the temporal cortex (TC), dorsal (DH), and ventral (VH) hippocampus in a lithium–pilocarpine model of temporal lobe epilepsy. The studies were performed two months after the seizure induction (chronic phase of the model). Ctrl—control group; Pilo—epileptic animals. Data are represented as individual values (circles and triangles) with the minimum and maximum (error bars), the sample median (horizontal line), and the first and third quartiles (boxes). Unpaired *t*-test: ** *p* < 0.01, *** *p* < 0.001.

The *Grm2* mRNA level in the dorsal hippocampus was increased in epileptic animals (Figure 5). This change was probably one of the compensatory mechanisms that were not expressed in the latent phase (Figure 2). However, *Grm3* gene expression was reduced in the temporal cortex.

**Figure 6:**
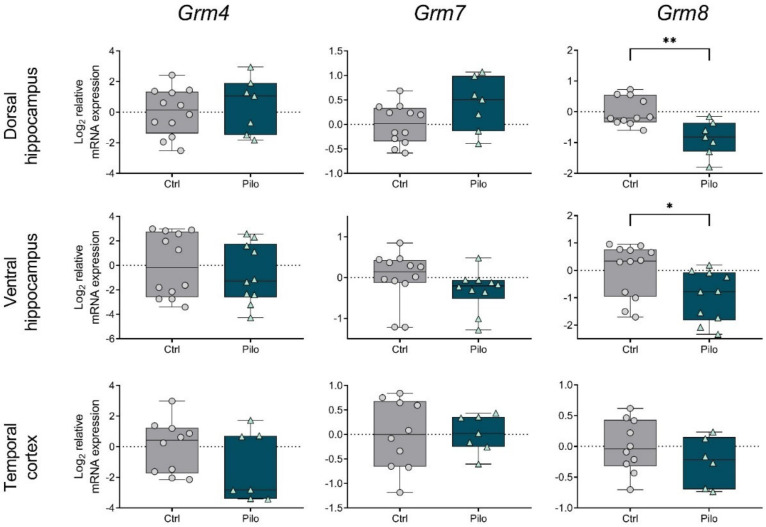
The relative mRNA expression of group III metabotropic glutamate receptors *Grm4, Grm7,* and *Grm8* in the temporal cortex (TC), dorsal (DH), and ventral (VH) hippocampus in a lithium–pilocarpine model of temporal lobe epilepsy. The studies were performed two months after the seizure induction (chronic phase of the model). Ctrl—control group; Pilo—epileptic animals. Data are represented as individual values (circles and triangles) with the minimum and maximum (error bars), the sample median (horizontal line), and the first and third quartiles (boxes). Unpaired *t*-test: * *p* < 0.05, ** *p* < 0.01.

As for group III of the mGluRs, a reduced expression of the *Grm8* gene persisted in the dorsal and ventral hippocampus, while the other changes found in the latent phase were not observed in epileptic animals (Figure 6).

## 3. Discussion

In the present work, we analyzed gene expression of the groups I (*Grm1*, *Grm5*), II (*Grm2*, *Grm3*), and III (*Grm4*, *Grm7*, *Grm8*) in the dorsal and ventral areas of the hippocampus and temporal cortex 3, 7, and 60 days after lithium–pilocarpine-induced seizures. The lithium–pilocarpine model was used in our study because it most closely reproduces the pathological changes occurring in patients with TLE. Pilocarpine, an M1 muscarinic cholinergic receptor agonist, causes an imbalance between excitatory and inhibitory transmission, resulting in status epilepticus (acute phase of the model) [16]. The mechanism for maintaining seizures then changes, as the muscarinic receptor antagonist atropine does not prevent the development of seizures [22]. The glutamatergic system, in particular the NMDA receptors, has been found to be involved in the maintenance of seizures [16,23,24]. This claim was supported by evidence of increased glutamate levels in the hippocampus after the onset of seizures [24]. After a latent period lasting several days or weeks, the animals exhibited spontaneous seizures (chronic phase) [16]. Subsequently, experimental animals showed impairments in memory and behavior [25,26] that were accompanied by neurodegenerative processes in the temporal cortex, amygdala, hippocampus, and some other brain regions [16], similar to lesions in temporal lobe epilepsy patients [27]. Changes in mGluR gene expressions and the role of these changes in epileptogenesis are still poorly understood. This study revealed changes in mGluR expression that may be a factor in epileptogenesis and changes specific to the epileptic brain. In addition, in this study, we selected and optimized multiplex test systems for the analysis of mGluR gene expressions. 

Changes in mGluR mRNA production differed in the latent and chronic phases of the model. During the latent phase, changes in the gene expression of mGluRs from groups I and III were detected, which could contribute to epileptogenesis. In particular, increased expression of the mGluR5 gene was found in both studied hippocampus regions, but not in the temporal cortex. This result coincided with data obtained by immunohistochemistry in rats following seizures induced by electrical stimulation [12]. Patients with temporal lobe epilepsy also had increased mGluR5 immunoreactivity [14] and protein levels [28] in the hippocampus. 

In contrast, another study in Wistar rats using pilocarpine-induced status epilepticus found a decrease in mGluR5 expression at the mRNA and protein levels [29]. The differences in the obtained results may have been due to the different ages of the animals and the seizure-induction protocol. During the chronic phase of the model, we found that revealed changes returned to control values in the hippocampus, while mGluR5 (and mGluR1) gene expression decreased in the temporal cortex. This was probably one of the compensatory mechanisms that reduced overexcitation.

We did not detect changes in the expression of group II mGluR genes during the latent phase. A study by Garrido-Sanabria et al. revealed downregulation of mGluR2 and mGluR3 expression in the hippocampus and cortex in a pilocarpine model 24 h after seizure induction [15]. We did not analyze expression at the acute phase; perhaps, by day 3, these possible changes were no longer detectable. 

The study using the rat model in which seizures were induced by electrical stimulation found increased mGluR2/3 protein levels in the hippocampus one week after the seizures, which persisted for up to 3 months [12]. Although we did not detect such changes in mRNA levels during the latent phase, an increase in mGluR2 was revealed in the dorsal hippocampus during the chronic phase in epileptic animals. In contrast, the temporal cortex showed decreased expression of the gene encoding mGluR3 in the chronic phase of the model. 

Gene expression of mGluRs from group III was significantly reduced in all studied regions during the latent phase of the lithium–pilocarpine model. A decrease in *Grm8* mRNA levels in both hippocampus regions was also detected in the chronic phase. A pilocarpine model showed a decrease in *Grm4* mRNA levels in the rat hippocampus [30]. However, the opposite change in *Grm8* expression was detected in this work. The mGluR4 knockout mice showed a significant increase in the severity of pilocarpine-induced seizures [31]. In this regard, perhaps the decreased *Grm4* expression we identified in the temporal cortex during the latent phase was most significant for epileptogenesis. In addition, the reduced expression of *Grm7* and *Grm8* that we found in all studied regions may also contribute to epilepsy development. In addition, *Grm8* gene expression remained reduced during the chronic phase in the dorsal area of the hippocampus.

A comparison of our mGluR mRNA production data with those obtained by other scientists revealed that the mRNA and protein levels did not always vary equally. For example, in a study by Aronica et al., mGluR2/3 protein levels were enhanced one week after seizures and remained reduced after 3 months [12]. However, in our study, an increase in mGluR2 gene expression was observed only during the chronic phase. Alterations in mRNA expression play an essential role in determining most changes in protein levels [32]. However, mismatches between changes in mRNA and protein levels can be detected in various pathological conditions, depending on the time of analysis and the detection method [33]. This indicates the importance of additional protein expression assessment studies to better understand the changes that occur to mGluRs during epileptogenesis.

Changes in the expression of genes encoding subunits of ionotropic glutamate NMDA and α-amino-3-hydroxy-5-methyl-4-isoxazolepropionic acid (AMPA) receptors, which may contribute to epileptogenesis, have previously been identified in our laboratory [34]. These data were also confirmed in this model by other researchers [35,36,37,38]. Thus, epileptogenesis is closely linked to abnormalities in the glutamatergic system [39]. However, severe side effects caused by NMDA receptor blockers limit the use of such drugs in the clinic. The use of mGluR ligands for epilepsy therapy may be a more promising approach. However, there are currently insufficient data for the use of pharmacological agents acting on mGluRs.

The obtained complex data on mGluR expression can be further used to select the most appropriate therapy for epilepsy [40] and prevention of epilepsy in risk groups. The use of agonists and antagonists of these receptors has long been regarded as a promising line of therapy [41]. Modulation of group I receptors has shown encouraging results. MPEP and MTEP, antagonists of group I mGluRs, provided a neuroprotective effect in models of epilepsy [42,43]. A study performed in our laboratory also showed the neuroprotective effect of MTEP, but administration of this antagonist did not prevent the development of epilepsy [44]. Simultaneously, activation of mGluR5 provided functional recovery after brain injury and reduced neuroinflammation in virus-induced epilepsy [45,46]. 

The effects of modulation of group II and III mGluRs are the least studied. In cultures of mouse cortical neurons, it was shown that positive modulation of mGluR4 had a neuroprotective effect, especially when applied with the mGluR1 antagonist [47]. However, a positive allosteric modulator of mGluR4 demonstrated a proconvulsant effect in models of epilepsy [48]. 

Overexpression of mGluR7 reduced seizure severity in kainate-induced epilepsy, whereas antagonism of mGluR7 increased epileptic seizures [49]. In addition, AMN082, the allosteric agonist of mGluR7, provided neuroprotective and glioprotective effects in glia, neuronal, and neuronal–glia cell cultures after various harmful stimuli [50]. The benefits of using an agonist of this receptor, LSP2-9166, also was confirmed by Girard and colleagues using the pentylenetetrazole kindling model [51].

Thus, this study extended our knowledge of the expression of mGluR genes at different stages of epileptogenesis. According to our findings, the use of group III mGluR agonists in combination with other pharmacological agents may be a promising approach to the prevention of epilepsy. 

## 4. Materials and Methods

### 4.1. Animals

The study was performed on 7–8-week-old male Wistar rats. The experiments involved 70 rats and were carried out following the Rules of Animal Care and Use Committee of the Sechenov Institute of Evolutionary Physiology and Biochemistry of the RAS and the EU Directive 2010/63/EU for animal experiments. The animals were kept under standard conditions with free access to water and feed, with natural daylight hours. The rats were randomly placed in groups. Animals from the same litter were placed in different groups to avoid the influence of genetic factors.

### 4.2. Lithium–Pilocarpine Model

The lithium–pilocarpine model of temporal lobe epilepsy was used in this study. The effectiveness of the used model protocol was demonstrated in our earlier research [52]. One day before pilocarpine injection, rats were injected with lithium chloride (intraperitoneally (i.p.), 127 mg/kg; Sigma-Aldrich, St. Louis, MO, USA). Then, one hour before pilocarpine administration, (−)-scopolamine methyl bromide (1 mg/kg, i.p.; Sigma-Aldrich) was injected to suppress the peripheral muscarinic receptors. Pilocarpine (20–40 mg/kg; Sigma-Aldrich) was given in 2–4 injections (10 mg/kg with 30 min intervals) until seizures achieved grade 4 on the Racine scale [21]. Seizures were stopped with diazepam (10 mg/kg, i.p., Sigma-Aldrich) after 75 min. For the first week after the seizures, rats were injected daily with 5% glucose solution (2 mL, subcutaneously). Animals were fed wet food to improve survival.

### 4.3. Spontaneous Recurrent Seizure Registration

Two months after seizure induction, we made a video recording of the rats’ free behavior to evaluate the presence of spontaneous recurrent seizures. For this, each rat was placed into a transparent cage (30 cm × 30 cm) with water and food for 3 consecutive days and videotaped every day for 12–16 h (for a total of 40 h). Only rats with at least one episode of motor seizures were included in the experimental group (*n* = 10).

### 4.4. Reverse Transcription Followed by Quantitative Real-Time PCR (RT-qPCR)

Rats were decapitated at 3, 7, and 60 days after seizures. The brain was quickly isolated and frozen at −80 °C. An OTF5000 freezing microtome (Bright Instruments, Luton, UK) was used to dissect the temporal cortex (TC), dorsal (DH), and ventral (VH) hippocampus according to rat brain atlas [53]. We isolated total RNA using the ExtractRNA reagent (Evrogen, Moscow, Russia). Before reverse transcription, RNA samples were treated with 1 unit of RQ1 DNAse (Promega, Madison, WI, USA). Then, the RNA was precipitated with LiCl. RNA concentration and purity were determined as described previously [44].

The cDNA was synthesized from 1 μg (VH) or 2 μg (TC, DH) of total RNA using oligo-dT (0.5 µg per 1 µg of RNA), 9-mer random (0.25 µg per 1 µg of RNA) primers (DNA Synthesis Ltd., Moscow, Russia), and M-MLV reverse transcriptase (100 units per 1 µg of RNA; Promega, Madison, WI, USA). Reverse transcription was performed according to the manufacturer’s protocol. The obtained cDNA samples were diluted 10-fold before the PCR. The qPCR was carried out in a total volume of 6 µL with 0.8 µL of cDNA. A total of 0.5 units of TaqM-polymerase was used for the reaction (Alkor Bio, St. Petersburg, Russia). Specific hydrolysis (TaqMan) probes and forward and reverse primers are shown in Appendix A, Table A1. Nucleotides were synthesized at DNA Synthesis Ltd. (Moscow, Russia). The primer and probe sequences were produced by the Primer Blast software (https://www.ncbi.nlm.nih.gov/tools/primer-blast accessed on 1 February 2022) against cDNA sequences of metabotropic receptor genes obtained from the National Center for Biotechnology Information (NCBI) RefSeq database. In the cases of designing probes for previously described primer pairs, we used Primer3Plus software (https://primer3plus.com/cgi-bin/dev/primer3plus.cgi accessed on 1 February 2022) followed by nucleotide BLAST specificity checking (https://blast.ncbi.nlm.nih.gov/Blast.cgi?PROGRAM=blastn&PAGE_TYPE=BlastSearch&LINK_LOC=blasthome accessed on 1 February 2022). The main parameters for primer design were set as follows: (1) the product size ranged from 50 to 180 nucleotides; (2) the annealing temperature was 57–63 °C (optimally 60 °C); and (3) the annealing temperature difference was limited to 3 °C. The main requirements for the probe design were set as follows: (1) a length of 18–27 nucleotides; (2) a melting temperature of the probe exceeding the melting temperature of the primers by 5–10 °C; (3) a GC content of 20–80%, and (4) the G nucleotide was avoided at the 5′ end, as it may quench the fluorescent signal.

The qPCR tests for mGluR genes were performed as multiplexes (*Grm1* + *Grm3* + *Grm5*, *Grm2* + *Grm7* + *Grm8*). Only the PCR reaction for *Grm4* was carried out as a singleplex. Housekeeping gene multiplexes (*Actb* + *Gapdh* + *B2m*, *Rpl13a* + *Ppia* + *Sdha*, and *Hprt1* + *Pgk1* + *Ywhaz*) were used for expression analysis according to a protocol described earlier [54]. The efficiency of the qPCR for mGluR genes was checked by the serial dilution method [55]. All reactions showed optimal efficiencies within the 90–105% range (see Appendix A, Figure A1). The qPCR was carried out in a C1000 Touch thermal cycler combined with a CFX384 Touch Real-Time PCR Detection System (BioRad, Hercules, CA, USA) in triplicates. The relative expression of metabotropic glutamate receptor genes was normalized to *Pgk1*, *Ywhaz*, *Gapdh* (3 and 7 days) and *Gapdh*, *Ywhaz*, *Hprt1* (60 days) in DH; *Gapdh*, *Ppia, Ywhaz* (3 and 7 days) and *Pgk1*, *Actb*, *Hprt1* (60 days) in VH; and *Gapdh, Pgk1*, *Hprt1* (3 and 7 days) and *Actb, Pgk1, Ywhaz* (60 days) in TC, and measured using the 2^−ΔΔCt^ method, as described elsewhere [56]. Reference genes were selected using the RefFinder online tool (https://www.heartcure.com.au/reffinder accessed on 1 February 2022).

### 4.5. Statistical Analysis

We used IBM SPSS Statistics 23 (IBM, Armonk, NY, USA) and GraphPad Prism 8.0.1 (GraphPad Software, San Diego, CA, USA) software for statistical analysis. We checked the normality of the sample using the Shapiro–Wilk test. Leven’s test was applied to check the equality of variance. Outliers were excluded using the quartile method. Two-way ANOVA with Sidak’s post hoc tests or unpaired t-tests were used for means comparisons. 

The differences were considered to be statistically significant at *p* ≤ 0.05. All data were represented as individual values with the minimum, the maximum, the sample median, and the first and third quartiles.

## Data Availability

The data presented in this study are available upon request from the corresponding author.

## Appendix A

**Table A1:** Primers and probes used in RT-qPCR.

Gene Symbol RefSeq Accession Number	Nucleotide Sequences (Forward, Reverse, TaqMan Probe)	Final Primer and Probe Concentrations (nM)	Reference
*Grm1* NM_001114330.1	GGGAATGCCAAGAAGAGGCAG CTGCAGTGTGGGGGTTTTCAA FAM-GCCCAGCAGCCAGTGTCCGTCGGC-BHQ1	400 200	This work
*Grm2* NM_001105711.1	TCCAGTGATTATCGGGTGCAG AACTTGGGTGCAAAGAGGCA FAM-TGCGTGTCCGTCAGCCTCAGTGGCT-BHQ1	200 100	This work
*Grm3* NM_001105712.1	CAGGAGTTGACGGTGCGAG GCCTGTCCTTCAGATAAGGGAG ROX-TCGGTGACGGGCTCTTTCAGCCCAA-BHQ2	200 200	This work
*Grm4* NM_022666.1	GGCAGTGCGAGCAGCTAAGG CCGGTCACTCCTACCAACCG FAM-CTCCCTGAGCTCCCCCGGAGCAGC-BHQ1	200 150	This work
*Grm5* NM_017012.1	ATGCATGTAGGAGACGGCAA TTTCCGTTGGAGCTTAGGGTTT HEX-CGTCCGCTGCCAGCAGATCCAGCA-BHQ2	400 200	[57] (primers); this work (probe)
*Grm7* NM_031040.1	CCAGACAACAAACACAACCAACC GCGTTCCCTTCTGTGTCTTCTTC HEX-TGCAGTGGGGCAAAGGAGTCCGAG-BHQ2	200 100	[58] (primers); this work (probe)
*Grm8* NM_022202.1	TCATCGGGCACTGGACAAAT CACGGTTTTCTTCCTCTCCCC ROX-TGTCTGCAGCCTGCCGTGCAAGCCC-BHQ2	300 100	This work
*Actb* NM_031144	TGTCACCAACTGGGACGATA GGGGTGTTGAAGGTCTCAAA FAM-CGTGTGGCCCCTGAGGAGCAC-BHQ1	200 200	[59] (primers); [54] (probe)
*Gapdh* NM_017008	TGCACCACCAACTGCTTAG GGATGCAGGGATGATGTTC R6G-ATCACGCCACAGCTTTCCAGAGGG-BHQ2	200 100	[60]
*B2m* NM_012512	TGCCATTCAGAAAACTCCCC GAGGAAGTTGGGCTTCCCATT ROX-ATTCAAGTGTACTCTCGCCATCCACCG-BHQ1	200 100	[61]
*Rpl13a* NM_173340	GGATCCCTCCACCCTATGACA CTGGTACTTCCACCCGACCTC FAM-CTGCCCTCAAGGTTGTGCGGCT-BHQ1	200 100	[62] (primers); [54] (probe)
*Sdha* NM_130428	AGACGTTTGACAGGGGAATG TCATCAATCCGCACCTTGTA R6G-ACCTGGTGGAGACGCTGGAGCT-BHQ2	200 100	[63] (primers); [54] (probe)
*Ppia* NM_017101	AGGATTCATGTGCCAGGGTG CTCAGTCTTGGCAGTGCAGA ROX-CACGCCATAATGGCACTGGTGGCA-BHQ1	200 100	[36]
*Hprt1* NM_012583	TCCTCAGACCGCTTTTCCCGC TCATCATCACTAATCACGACGCTGG FAM-CCGACCGGTTCTGTCATGTCGACCCT-BHQ1	200 100	[64] (primers); [54] (probe)
*Pgk1* NM_053291	ATGCAAAGACTGGCCAAGCTAC AGCCACAGCCTCAGCATATTTC R6G-TGCTGGCTGGATGGGCTTGGA-BHQ2	200 100	[65] (primers); [54] (probe)
*Ywhaz* NM_013011	GATGAAGCCATTGCTGAACTTG GTCTCCTTGGGTATCCGATGTC ROX-TGAAGAGTCGTACAAAGACAGCACGC-BHQ1	200 100	[65] (primers); [54] (probe)
